# Research trends and knowledge mapping of transcranial direct current stimulation in depression: a bibliometric study based on web of science, Scopus, and PubMed (2000-2025)

**DOI:** 10.3389/fpsyt.2026.1805441

**Published:** 2026-05-01

**Authors:** Dongyu Feng, Liuyin Jin, Minsha Mei, HongWei Yuan

**Affiliations:** 1 School of Basic Medical Sciences, Zhejiang Chinese Medical University, Hangzhou, Zhejiang, China; 2 The Second People’s Hospital of Lishui, Lishui, China

**Keywords:** bibliometrics, citation burst, depressive disorder, functional connectivity, neuromodulation, scientometrics

## Abstract

**Background:**

Depressive disorders are clinically heterogeneous and mechanistically complex psychiatric conditions. Transcranial direct current stimulation (tDCS), a key non-invasive neuromodulation technique, has expanded rapidly in both therapeutic application and mechanistic research. However, the field is marked by rapid publication growth, thematic diversity, and variability in evidence quality. A systematic quantitative synthesis is therefore needed to map the research landscape, identify hotspots, and inform future directions.

**Methods:**

A systematic search was conducted for English-language publications in the Web of Science Core Collection (WoSCC), Scopus, and PubMed using the terms (“Transcranial direct current stimulation” OR “tDCS”) AND (“depression” OR “major depressive disorder” OR “depressive disorder” OR “MDD”). Only articles and reviews were included. Records from 2026 and non-research publications, including conference abstracts, editorials, letters, news items, and errata, were excluded. Deduplication was performed using DOI-based matching followed by title-assisted matching. Bibliometrix (R), VOSviewer, and CiteSpace were used to analyze publication trends, contributions by countries/regions, institutions, authors, and journals, collaboration networks, keyword co-occurrence, thematic clustering, and burst terms. Citation analysis was based on WoSCC data only.

**Results:**

Research on tDCS for depression showed sustained growth, with marked acceleration after 2020 and a peak in 2024. The United States, Germany, and Brazil occupied central positions in both productivity and international collaboration, with the United States ranking first in publication volume. Major research hubs included the Universidade de São Paulo, the University of Toronto, and Harvard University, while Brain Stimulation, Journal of Affective Disorders, and Frontiers in Psychiatry were the leading publication venues. Highly cited studies mainly focused on neurophysiological mechanisms, pivotal randomized controlled trials, and evidence-based guidelines. Keyword analyses indicated a shift from early attention to cortical excitability, safety, and short-term efficacy toward a more integrated framework involving prefrontal-targeted stimulation, cognitive function, functional connectivity, treatment outcomes, and cross-disorder applications.

**Conclusion:**

tDCS research in depression is entering a multidimensional and interdisciplinary phase, with increasing emphasis on network-level mechanisms and precision intervention. Functional connectivity is emerging as a potential biomarker for patient stratification and outcome prediction. Further progress depends on multicenter standardization, reproducible analytic pipelines, and high-quality comparative effectiveness research.

## 1 Introduction

Major Depressive Disorder (MDD) is a highly prevalent and debilitating psychiatric condition, clinically defined by persistent dysphoria, anhedonia, and volitional suppression, frequently compounded by cognitive deficits and neurovegetative dysregulation (1, 2). Despite significant pharmacological and psychotherapeutic advances, routine clinical practice is hindered by the substantial heterogeneity of treatment responses, latency in therapeutic onset, and high recurrence rates (3); A critical limitation in current psychiatry lies in the diagnostic paradigm, which relies on a composite framework of “categorical diagnosis + psychometric scaling + functional assessment.” While DSM/ICD-guided interviews and instruments such as the HAMD or MADRS provide necessary standardization, they are inherently sensitive to subjective reporting and state fluctuations. This phenotypic complexity often masks divergent underlying biological substrates under a single diagnostic label. Consequently, the establishment of objective, quantifiable biomarkers capable of facilitating patient stratification and response prediction represents an urgent, unmet scientific exigency.

Transcranial direct current stimulation (tDCS) has emerged as a promising non-invasive neuromodulation strategy, valued for its favorable safety profile, cost-effectiveness, and ease of administration (4, 5). Mechanistically, tDCS delivers sub-threshold direct currents via scalp electrodes to modulate cortical excitability and synaptic neuroplasticity, thereby influencing both local neuronal activity and large-scale functional networks (6–8). Theoretical models have progressively evolved from simplistic “anodal-excitation/cathodal-inhibition” dichotomies to sophisticated network-level hypotheses, positing that tDCS ameliorates depressive symptoms by recalibrating the functional coupling between prefrontal cognitive control regions and limbic emotion-regulation circuitry (9–11). Despite this progress, the literature is replete with variability in effect sizes and individual responsiveness. Furthermore, critical parameters—including montage targeting, dosage density, and treatment duration—lack universal standardization (12, 13).

In recent years, bibliometrics has been widely leveraged as a quantitative methodology grounded in publication, citation, and text-mining data to profile scientific landscapes and knowledge evolution across interdisciplinary domains (14, 15). By modeling the relationships among authors, institutions, countries, journals, and keywords, bibliometric analyses not only provide descriptive statistics (e.g., productivity and citation frequency) but also reveal collaboration structures, intellectual bases, and research hotspots via network and clustering techniques, and further detect emerging frontiers and thematic migration through citation burst analysis (16). For a rapidly expanding field such as tDCS and depression—where research paradigms iterate quickly and evidence frameworks are continuously updated—bibliometrics offers a macro, systematic, and reproducible vantage point to address key questions: how the field evolves, where hotspots migrate, who the principal contributors are, what constitutes the core evidence, and where future research should concentrate (17, 18).

Accordingly, the present study focuses on the literature concerning “tDCS/neuromodulation and depression,” integrates bibliographic records from the Web of Science Core Collection (WoSCC), Scopus, and PubMed, and conducts bibliometric and visualization analyses using bibliometrix (R), VOSviewer, and CiteSpace. The analyses encompass: (i) publication trends and growth characteristics; (ii) productivity and collaboration networks across countries/regions, institutions, and authors; (iii) core journals and high-impact publications (global and local citations); and (iv) keyword co-occurrence, thematic clustering, and citation bursts to elucidate knowledge structure and frontier evolution. Through these analyses, we aim to delineate the field’s developmental trajectory from an evidence-integration and science-mapping perspective, identify pivotal themes and plausible future directions, and provide a data-driven reference framework for mechanistic research, parameter optimization, and translational pathway design.

This study attempts a relatively systematic synthesis of research on “tDCS/neuromodulation and depression” from a bibliometric and visualization perspective. We integrated bibliographic records from the WoSCC, Scopus, and PubMed; completed multi-database retrieval, deduplication, and standardized normalization to assemble a comprehensive dataset; and leveraged bibliometrix (R), VOSviewer, and CiteSpace to analyze publication trends, country/institution/author collaboration networks, distributions of core journals and high-impact papers, as well as keyword co-occurrence, thematic clustering, and citation bursts. Collectively, these efforts are intended to provide an integrative overview of the research landscape and thematic evolution, and to offer leads for subsequent discussions on mechanisms, parameter optimization, and clinical translation.

## 2 Methods

### 2.1 Data sources and search strategy

A systematic search was conducted in the WoSCC, Scopus, and PubMed databases to identify publications related to tDCS and depression. To ensure topic specificity and avoid conceptual contamination, only tDCS-specific terms were used, while broader neuromodulation terms such as “noninvasive brain stimulation” and “neuromodulation” were not included (19–21). The search strategies were as follows: TS=(“transcranial direct current stimulation” OR “tDCS”) AND TS=(“depression” OR “major depressive disorder” OR “depressive disorder” OR “MDD”). Scopus: TITLE-ABS-KEY(“transcranial direct current stimulation” OR “tDCS”) AND TITLE-ABS-KEY(“depression” OR “major depressive disorder” OR “depressive disorder” OR “MDD”). PubMed: (“transcranial direct current stimulation”[Title/Abstract] OR tDCS[Title/Abstract]) AND (depression[Title/Abstract] OR “major depressive disorder”[Title/Abstract] OR “depressive disorder”[Title/Abstract] OR MDD[Title/Abstract]). The overall time window for analysis was defined from the earliest available publications to the end of 2025. Considering that records from 2026 were not yet fully indexed, including them might introduce time-truncation bias; therefore, they were excluded from the final analysis (22, 23).

### 2.2 Record export and dataset integration

Following retrieval, bibliographic data were exported from each database: Web of Science records were exported as “Full record and cited references,” Scopus records in BibTeX format, and PubMed as Citation Manager (.nbib). All records were then imported into R (version 4.5.2) and converted into a unified bibliographic data structure using the bibliometrix package to facilitate cross-database integration. At the initial stage, records from the three databases were merged without performing automatic cross-database deduplication, allowing subsequent steps—such as year filtering, document type selection, and deduplication—to be conducted in a transparent and reproducible manner.

### 2.3 Year filtering, document type selection, and deduplication

**Figure 1:**
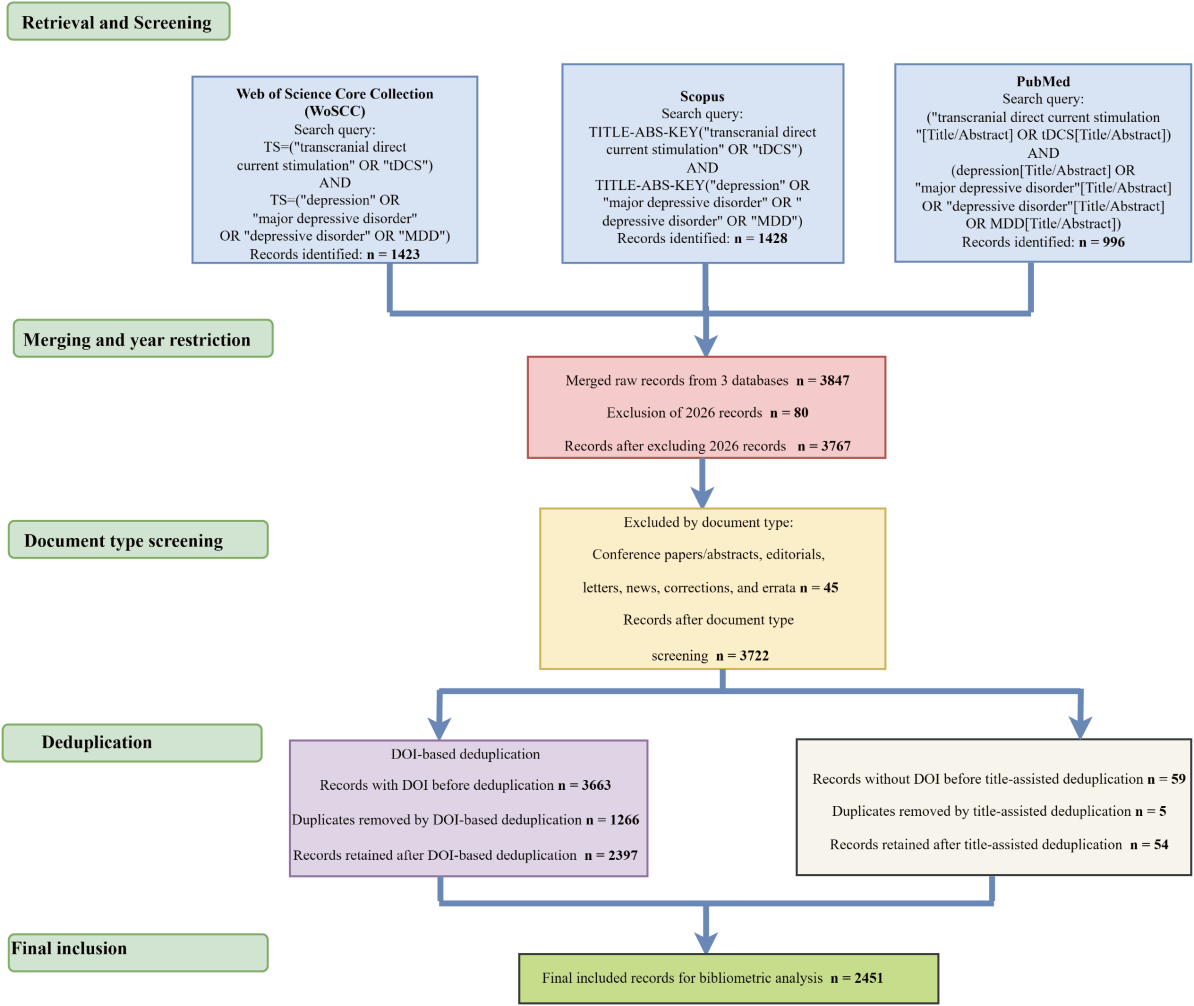
Literature retrieval, screening, and selection process. Records were retrieved from Web of Science Core Collection, Scopus, and PubMed using predefined search strategies. After merging datasets and removing duplicates (primarily based on DOI, with title-assisted deduplication for records without DOI), records were screened by document type. Conference papers, abstracts, editorials, letters, news, corrections, and errata were excluded. A total of 2,451 records were finally included for bibliometric analysis.

In the merged dataset, records published in 2026 were first excluded. This step was performed separately because data from that year were still undergoing indexing, and their inclusion could affect the stability of temporal trend and structural analyses. Subsequently, document types were filtered, retaining only articles and reviews, while excluding conference papers or abstracts, editorials, letters, news items, errata, and other non-research records. Deduplication was conducted using a two-step strategy: “DOI-first, title-assisted.” First, DOI fields were standardized by unifying letter case and removing prefixes and extra spaces, followed by exact matching based on standardized DOIs. For records lacking DOI information, title-assisted deduplication was applied. Specifically, titles were normalized in terms of case, punctuation, and spacing, and duplicate records were identified through title matching. The final cleaned dataset was used for subsequent bibliometric analyses. The overall process is illustrated in **Figure 1**.

### 2.4 Bibliometric analyses

Using the cleaned final dataset, bibliometric analyses were conducted to characterize productivity patterns and the research landscape, including:

1. annual publication trajectories;
2. country/region distributions (reporting publication counts only, without citation-based indicators);
3. institution-level profiling, including identification of high-output institutions;
4. author-level productivity profiling and identification of highly productive authors;
5. journal distributions and publication characteristics.

### 2.5 Keyword co-occurrence and thematic evolution analysis

To identify research hotspots and their evolutionary patterns in studies on tDCS for depression, keyword analyses were conducted using R, VOSviewer, and CiteSpace. Keywords were derived from author keywords (DE), index keywords (ID), and available subject-heading fields (e.g., MH). First, keyword preprocessing was performed in R on the merged and deduplicated final dataset, including the removal of general demographic and methodological stop words ((such as “human(s)”, “male”, “female”, “adult”, “review”, and “controlled study”), standardization of synonyms and abbreviations (e.g., unifying “transcranial direct current stimulation” and “tDCS”, as well as “depression”, “major depressive disorder”, “MDD”, and “depressive disorder(s)”), and harmonization of structural differences across DE, ID, and MH fields. The cleaned keyword dataset was used for the main-text analyses, whereas the uncleaned raw keyword results were retained in the **Supplementary Materials**.

For keyword co-occurrence network construction, a keyword co-occurrence matrix was first generated in R based on document–keyword relationships and then exported as a Pajek.net network file. To improve network interpretability and reduce interference from low-frequency noise, two thresholds were applied during network construction: the minimum keyword occurrence threshold was set at 40 (MIN_OCC = 40), meaning that only keywords appearing at least 40 times were included; the minimum co-occurrence edge-weight threshold was set at 5 (MIN_EDGE = 5), meaning that only edges with a co-occurrence weight of at least 5 were retained. The resulting.net file was then imported into VOSviewer for network layout, cluster identification, and visualization. Therefore, node selection, co-occurrence construction, and threshold control in the keyword network were primarily performed in R, whereas VOSviewer was mainly used for network mapping and cluster visualization rather than for the direct generation of raw co-occurrence data. In addition to the keyword network map, keyword word clouds, treemaps, and keyword frequency bar charts were further generated in R, and the underlying source data were exported simultaneously to ensure consistency and reproducibility between the graphical outputs and the underlying data.

In CiteSpace, to simultaneously capture the long-term evolutionary trajectory of the field and recent research frontiers, two time windows were defined: 2000–2025 and 2015–2025. The former was used to present the overall thematic evolution since the earliest included publication, whereas the latter was used to focus on hotspot shifts and frontier changes over the past decade. The time slice was set to 1 year per slice. Term sources included titles, abstracts, author keywords (DE), and Keywords Plus (ID). Network link strength was set to cosine, and node selection was based on the g-index (k = 25). On this basis, keyword co-occurrence, burst-term detection, and thematic evolution visualization were conducted to compare similarities and differences between long-term evolutionary patterns and recent hotspot changes.

### 2.6 Institutional collaboration network analysis

Institutional collaboration network analysis was conducted using VOSviewer. The analysis type was set to Co-authorship, with Organizations selected as the unit of analysis, and the counting method set to full counting. The inclusion thresholds for institutions were defined as a minimum of 10 publications and at least 0 citations. To reduce the influence of large-scale multi-institutional collaborations on the network structure, the option “Ignore documents co-authored by a large number of organizations” was selected, and the maximum number of organizations per document was set to 25. Under these conditions, a total of 1,824 institutions were included in the candidate set, of which 65 met the threshold criteria and were included in the final visualization network.

### 2.7 Citation relationship analyses

#### 2.7.1 Data scope and analytic boundary

Citation relationship analyses (e.g., co-citation networks and citation-based metrics) require complete cited-reference fields. Because PubMed does not provide standardized cited-reference and citation metadata, all citation-relationship analyses in this study were confined to WoSCC data to preserve structural consistency and interpretability.

#### 2.7.2 Analytic procedures

From WoSCC records, citation counts and cited-reference lists were extracted to conduct: 1. Global Citations: the total number of citations a given publication has received within the entire WoSCC database, reflecting its overall academic impact in the broader scientific community; 2. Local Citations: the number of times a publication is cited by other articles within the dataset included in this study, reflecting its knowledge influence and central position within the specific research field of tDCS and depression.

#### 2.7.3 Notes on citation analyses and methodological consistency

Citation-relationship analyses are intended to characterize relative influence and structural position within scholarly networks rather than to directly appraise study quality. Given differences across databases in citation coverage and counting rules, and the incompleteness of PubMed citation metadata, we did not incorporate PubMed and Scopus into citation-relationship analyses to reduce bias from missingness and inconsistent counting. Accordingly, a modular strategy was adopted: the merged multi-database dataset (Web of Science, Scopus, and PubMed) was used to describe productivity patterns and thematic structure, whereas citation-relationship analyses (e.g., co-citation networks and citation metrics) were conducted exclusively on WoSCC data. This strategy preserves breadth of coverage while maintaining reliability and methodological coherence for citation analyses (24).

## 3 Results

### 3.1 Retrieval results

A total of 3,847 records were retrieved from the, Scopus, and PubMed databases, including 1,423 from WoSCC, 1,428 from Scopus, and 996 from PubMed. After merging the datasets, records published in 2026 were excluded (n = 80), leaving 3,767 records. Document type screening was then performed, and non-research records such as conference abstracts, editorials, letters, and errata were removed (n = 45), resulting in 3,722 records entering the deduplication process. Deduplication was performed using a two-step strategy of DOI-first matching and title-assisted verification. Among the 3,663 records with DOI information, 1,266 duplicates were identified and removed, leaving 2,397 records. Among the remaining 59 records without DOI information, 5 duplicates were removed through title comparison, leaving 54 records. Ultimately, 2,451 publications were included for subsequent bibliometric analyses (**Figure 1**).

### 3.2 Annual publication trends in tDCS research on depression

**Figure 2:**
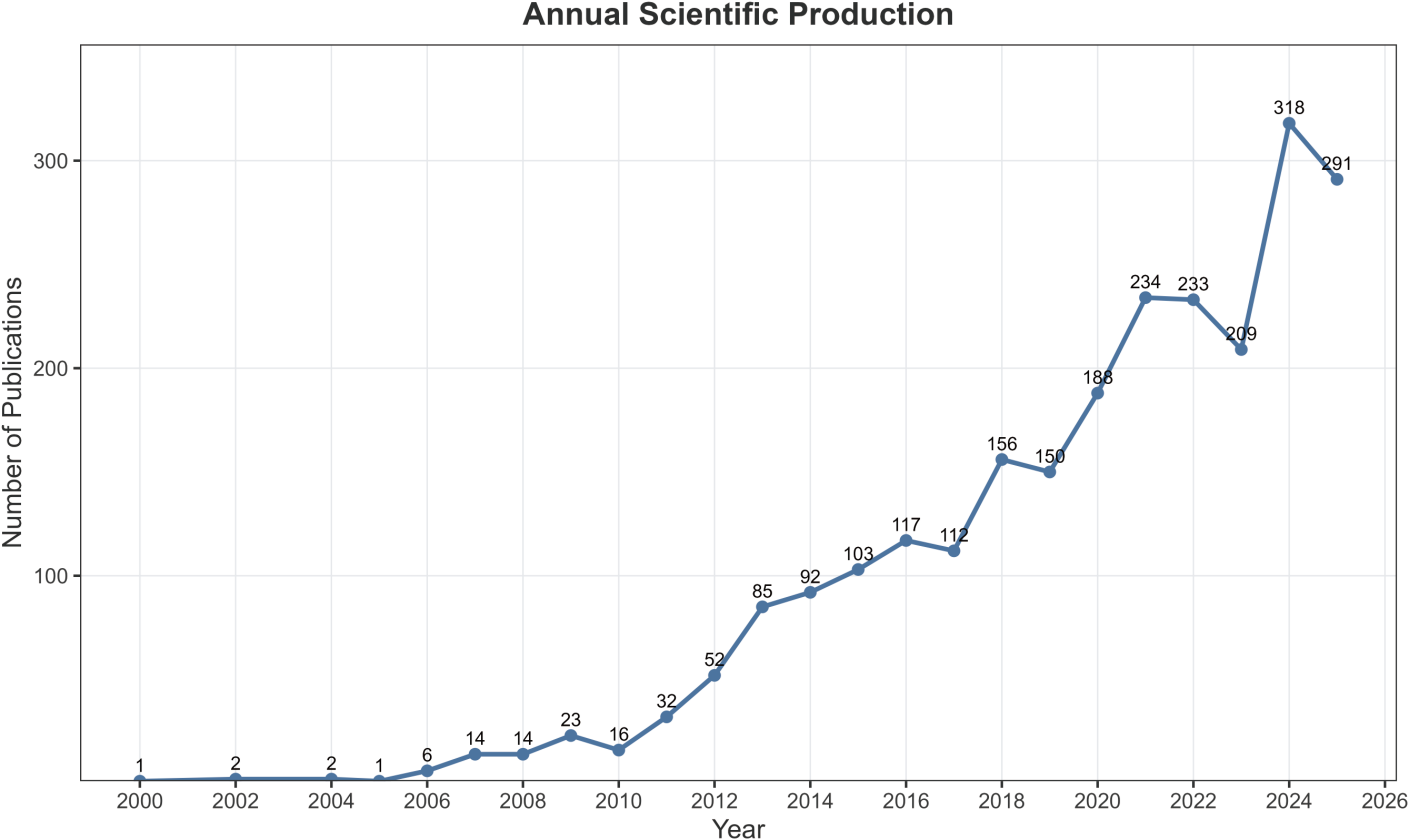
Annual scientific production of the included literature. The number of publications on transcranial direct current stimulation (tDCS) in depression increased steadily over time, with a marked acceleration after 2015 and a peak observed in recent years, indicating growing research interest and expansion of the field.

**Figure 2** shows the annual publication trends in studies on tDCS and depression from 2000 to 2025. Overall, the field exhibited a sustained upward trajectory, progressing from sporadic early exploration to steady growth and eventually entering a phase of rapid development. Between 2000 and 2005, annual publication output remained very low, at approximately 1–2 articles per year, indicating that the field was still in its initial exploratory stage. From 2006 onward, the number of related publications began to increase gradually (e.g., 6 in 2006, 14 in 2007, and 23 in 2009), reflecting growing academic interest in the topic. Between 2011 and 2015, publication output increased further (e.g., 32 in 2011, 52 in 2012, 85 in 2013, and 103 in 2015), indicating that the field had entered a relatively stable growth phase.

After 2016, research activity continued to rise. Although slight fluctuations were observed in some years (e.g., 112 in 2017, 150 in 2019, and 209 in 2023), the overall upward trend remained unchanged. Particularly after 2020, publication output accelerated markedly, with 188 publications in 2020, 234 in 2021, and 233 in 2022, reaching a peak of 318 in 2024 and remaining at a relatively high level in 2025 (291 publications). Overall, **Figure 2** indicates that research on tDCS for depression underwent a transition from an early exploratory phase to rapid development between 2000 and 2025 and has now become a continuously expanding research direction.

### 3.3 Country distribution and collaboration patterns

**Table 1** summarizes the productivity and collaboration characteristics of the top 25 countries in tDCS research on depression after merging variant country names. Overall, the United States dominated the field, with 1,060 publications, far exceeding all other countries. However, its number of single-country publications (SCP) was only 130, whereas the number of multiple-country publications (MCP) reached 930, corresponding to an MCP ratio of 87.7%, indicating that the United States was deeply embedded in the international collaboration network in this field. Germany ranked second with 450 publications and an MCP ratio of 70.7%, while Brazil ranked third with 373 publications and an MCP ratio of 83.6%, demonstrating strong cross-national collaboration. China ranked fourth with 360 publications and was one of the few leading countries characterized primarily by domestic production, with 232 SCPs and 128 MCPs, corresponding to an MCP ratio of only 35.6%. This indicates that research output from China relied more heavily on domestic teams. Italy (253 publications), Australia (244), the United Kingdom (225), and Canada (222) followed, all showing relatively high levels of international collaboration, with MCP ratios of 64.4%, 69.7%, 81.8%, and 74.3%, respectively. Notably, although the United Kingdom and Canada had slightly lower total publication volumes than Germany and Brazil, both exhibited strong international collaboration intensity, suggesting pronounced cross-national cooperation in their research activities. It is also noteworthy that several countries with relatively lower publication output showed even higher proportions of international collaboration. Portugal had the highest MCP ratio at 90.6%, followed by New Zealand (88.0%), Israel (87.5%), Belgium (85.7%), and Switzerland (85.5%). This pattern suggests that although these countries had smaller absolute output, their knowledge production relied more heavily on international collaboration networks. In contrast, India (31.8%), Poland (32.0%), China (35.6%), and South Korea (37.8%) had relatively low MCP ratios, indicating that their research outputs were more often produced independently by domestic institutions.

**Table 1:** Top 25 countries after merging duplicate country variants.

Rank	Country	Publications	SCP	MCP	MCP (%)
1	United States	1060	130	930	87.7
2	Germany	450	132	318	70.7
3	Brazil	373	61	312	83.6
4	China	360	232	128	35.6
5	Italy	253	90	163	64.4
6	Australia	244	74	170	69.7
7	United Kingdom	225	41	184	81.8
8	Canada	222	57	165	74.3
9	France	164	53	111	67.7
10	Belgium	147	21	126	85.7
11	Iran	140	66	74	52.9
12	Spain	113	24	89	78.8
13	South Korea	90	56	34	37.8
14	Netherlands	85	14	71	83.5
15	Japan	78	27	51	65.4
16	Switzerland	69	10	59	85.5
17	India	66	45	21	31.8
18	Taiwan	54	20	34	63.0
19	Portugal	32	3	29	90.6
20	Austria	29	6	23	79.3
21	Denmark	28	5	23	82.1
22	Turkey	26	14	12	46.2
23	New Zealand	25	3	22	88.0
24	Poland	25	17	8	32.0
25	Israel	24	3	21	87.5

Country variants were merged before ranking (e.g., USA/state-address entries to United States; Peoples R China/Pr China/Cn to China; England/Wales/UK to United Kingdom; Republic Of Korea to South Korea; The Netherlands to Netherlands). Non-country strings were removed.

Overall, **Table 1** shows that tDCS research on depression has developed a clear pattern of international collaboration. High-output countries such as the United States, Germany, and Brazil occupied central positions in the global research network, while some medium-output countries enhanced their academic participation and influence through a high proportion of international collaboration. This suggests that knowledge production in this field depends not only on the sustained contributions of a few highly productive countries but also benefits substantially from transnational collaborative networks.

### 3.4 Top 10 authors, journals, and institutions in the field of tDCS research on depression

**Figure 3:**
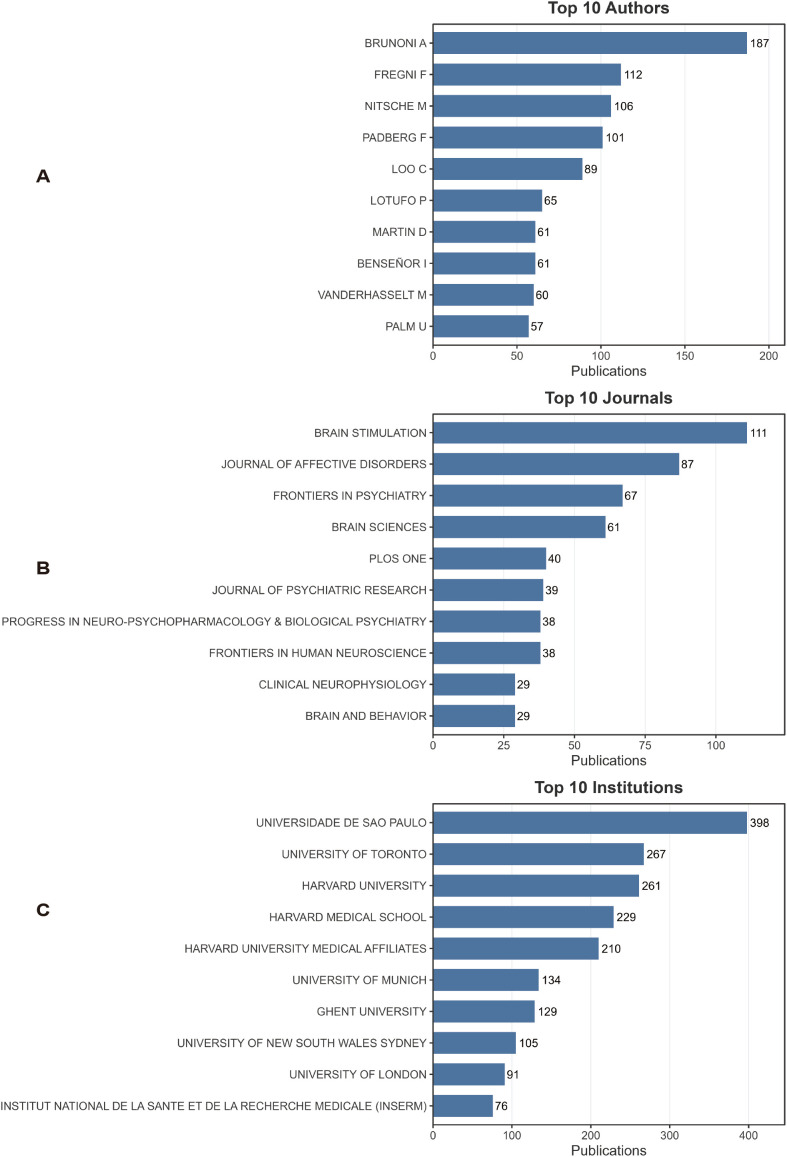
Top productive contributors and sources in the field. **(A)** Top 10 authors ranked by number of publications. **(B)** Top 10 journals publishing research in this field. **(C)** Top 10 institutions ranked by publication output. Bar length represents the number of publications. These results highlight the key contributors and core publication venues in the field.

**Figure 3** presents the top 10 authors (A), journals (B), and institutions (C) ranked by publication output in the field of tDCS research on depression. At the author level (**Figure 3A**), BRUNONI A ranked first with 187 publications, clearly ahead of all other scholars, and thus represented the most prominent core author in the field. He was followed by FREGNI F (112 publications), NITSCHE M (106), and PADBERG F (101), who together formed the main academic backbone of the field. Other authors, including LOO C (89), LOTUFO P (65), MARTIN D (61), BENSON I (61), VANDERHASSELT M (60), and PALM U (57), had relatively similar publication outputs, showing a pattern characterized by a highly prominent leading group and a relatively balanced second tier. This suggests that a relatively stable core author group has already formed in this research area.

In terms of journal distribution (**Figure 3B**), Brain Stimulation ranked first with 111 publications and was the most important outlet in this field. It was followed by Journal of Affective Disorders (87 publications) and Frontiers in Psychiatry (67). In addition, journals such as Brain Sciences (61), PLOS ONE (40), and Journal of Psychiatric Research (39) also showed relatively high publication volumes. Overall, research in this field was mainly concentrated in journals related to neuromodulation, psychiatry, and neuroscience, reflecting the interdisciplinary nature of tDCS research at the intersection of neurostimulation and psychiatric disorders.

At the institutional level (**Figure 3C**), Universidade de São Paulo ranked first with 398 publications, highlighting its outstanding research strength in this field. It was followed by the University of Toronto (267) and Harvard University (261). Notably, Harvard Medical School (229) and its affiliated institutions (210) also ranked among the leading contributors, indicating sustained and concentrated research output from the Harvard system. Other active institutions included the University of Munich (134), Ghent University (129), the University of New South Wales Sydney (105), the University of London (91), and INSERM (76). Overall, the institutional distribution showed a pattern dominated by high-level research institutions in Europe, North America, and parts of Latin America. Taken together, research on tDCS for depression has developed a stable academic network composed of core authors, leading journals, and highly productive institutions. A limited number of leading authors and institutions play dominant roles in knowledge production, while research findings are mainly published in journals related to neuromodulation and psychiatry.

### 3.5 Keyword co-occurrence and distribution of research hotspots

**Figure 4:**
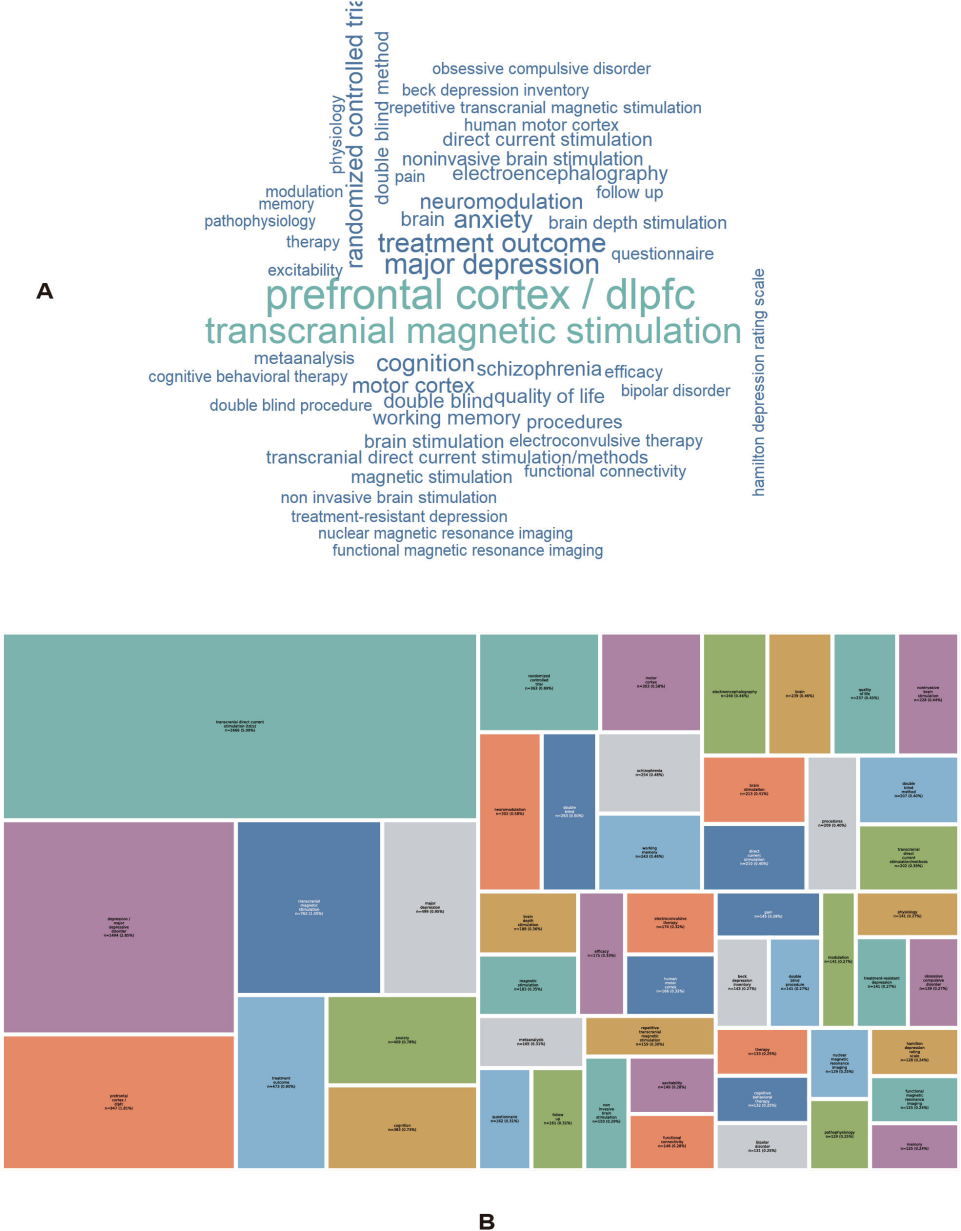
Keyword distribution and frequency. **(A)** Word cloud of keywords, where font size represents frequency of occurrence. **(B)** Treemap visualization of keyword frequencies, where rectangle size reflects the relative contribution of each keyword. Keywords were standardized prior to analysis. Visualization reflects thematic prominence rather than semantic relationships.

**Figure 4** illustrates the keyword distribution characteristics in tDCS research on depression, including a keyword word cloud (A) and a treemap (B). In the keyword word cloud (**Figure 4A**), the font size of each keyword reflects its frequency of occurrence. The most prominent high-frequency keywords were “prefrontal cortex/dorsolateral prefrontal cortex(dlPFC)” and “transcranial magnetic stimulation,” indicating that current research mainly focuses on the prefrontal cortex, especially the, as a key stimulation target, as well as on noninvasive brain stimulation techniques such as transcranial magnetic stimulation. In addition, keywords such as “major depression,” “treatment outcome,” and “anxiety” also occupied substantial weights, suggesting that research has mainly concentrated on the clinical efficacy of depression treatment and its relationship with emotional symptoms.

Several important associated directions could also be identified around these core themes. First, methodological keywords such as “randomized controlled trial”,”double blind” and “meta-analysis” indicate a relatively high level of evidence-based research in this field. Second, keywords related to neuromodulation and mechanism-oriented research, such as “neuromodulation”,”functional connectivity”,”electroencephalography,” and “magnetic resonance imaging,” suggest that research has gradually expanded from clinical efficacy evaluation to exploration of underlying neural mechanisms. Third, keywords related to cognition and functional outcomes, including “cognition,” “working memory,” and “quality of life,” reflect increasing attention to cognitive performance and quality of life.

The keyword treemap (**Figure 4B**) further represents keyword importance and relative weight through area size. The results show that terms related to noninvasive brain stimulation technologies (e.g., tDCS and TMS) and prefrontal cortex-related research occupied the largest areas, constituting the core framework of this field. Keywords such as “treatment outcome,” “major depression,” and “randomized controlled trial” also accounted for substantial proportions, further confirming the central role of clinical efficacy evaluation and high-quality study designs. In contrast, although many other keywords were present, their individual weights were relatively low, showing a pattern characterized by a prominent core theme and a long-tail distribution.

Overall, keyword analysis indicates that research on tDCS for depression has developed into a multilayered system centered on prefrontal-targeted neuromodulation, guided by clinical efficacy evaluation, and progressively extending toward neural mechanisms and functional outcomes.

### 3.6 Institutional collaboration and keyword co-occurrence networks based on VOSviewer

**Figure 5:**
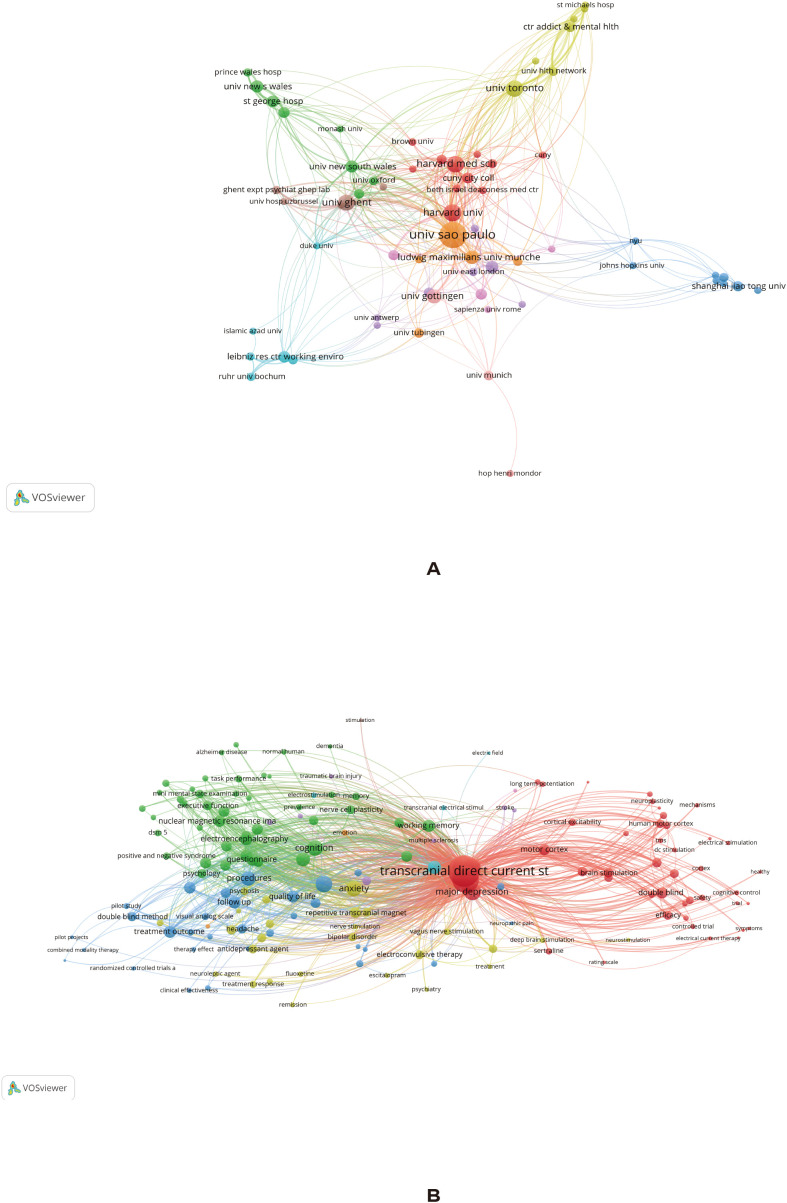
Collaboration and co-occurrence networks generated by VOSviewer. **(A)** Shows the institutional collaboration network, in which node size reflects publication output, link thickness reflects collaboration strength, and colors indicate clusters. **(B)** Shows the keyword co-occurrence network, in which node size reflects occurrence frequency and colors represent thematic groupings.

**Figure 5** presents the institutional collaboration network (A) and keyword co-occurrence network (B) in tDCS research on depression. In the institutional collaboration network (**Figure 5A**), different colors represent different collaboration clusters, node size reflects institutional publication output, and links represent collaborative relationships between institutions. From the overall structure, the field has formed several collaboration clusters centered on highly productive institutions. Institutions such as Universidade de São Paulo, Harvard University and its affiliated institutions, and the University of Toronto were located in the center of the network, with larger nodes and denser links, indicating their hub positions in the international collaboration network.

Extensive cross-connections among clusters suggest a high degree of international integration in this field. Specifically, institutions from Europe, North America, and Latin America constituted the main collaborative bodies and formed several interconnected core clusters, whereas some Asian institutions, including Chinese universities, were located relatively at the network periphery, with smaller node sizes and fewer connections, indicating room for improvement in their degree of international collaboration. Overall, the institutional network showed a structural pattern characterized by highly concentrated core institutions, close cross-national collaboration, and relatively scattered peripheral institutions.

In the keyword co-occurrence network (**Figure 5B**), node size represents keyword frequency, links represent co-occurrence relationships between keywords, and different colors represent different thematic clusters. The results show that ““ was located at the center of the network, linking multiple thematic clusters and serving as the core research focus of the field. Structurally, the keyword network can be broadly divided into several major research directions: (1) Core cluster of clinical treatment and depression-related research (red area), centered on keywords such as “major depression,” “treatment,” and “efficacy,” reflecting the application of tDCS in depression treatment and efficacy evaluation; (2) Cluster of neural mechanisms and brain function research (green area), including keywords such as “cognition,” “electroencephalography,” “functional connectivity,” and “brain,” indicating that research has gradually expanded toward neural mechanisms and brain functional regulation; (3) Cluster of clinical trials and study design (blue/yellow area), including keywords such as “randomized controlled trial,” “double blind,” and “treatment response,” suggesting that evidence-based medicine forms an important foundation of this field; (4) Extended cluster of psychiatric disorders, including terms such as “schizophrenia” and “anxiety,” suggesting that the application of tDCS has expanded from depression to other psychiatric disorders.

In addition, close interconnections among clusters suggest substantial overlap and integration across different research directions. Overall, the field has developed into a multidimensional research structure centered on tDCS, oriented toward clinical application, and characterized by parallel progress in mechanistic and methodological research.

### 3.7 Research hotspots revealed by CiteSpace keyword clustering and citation burst analysis

**Figure 6:**
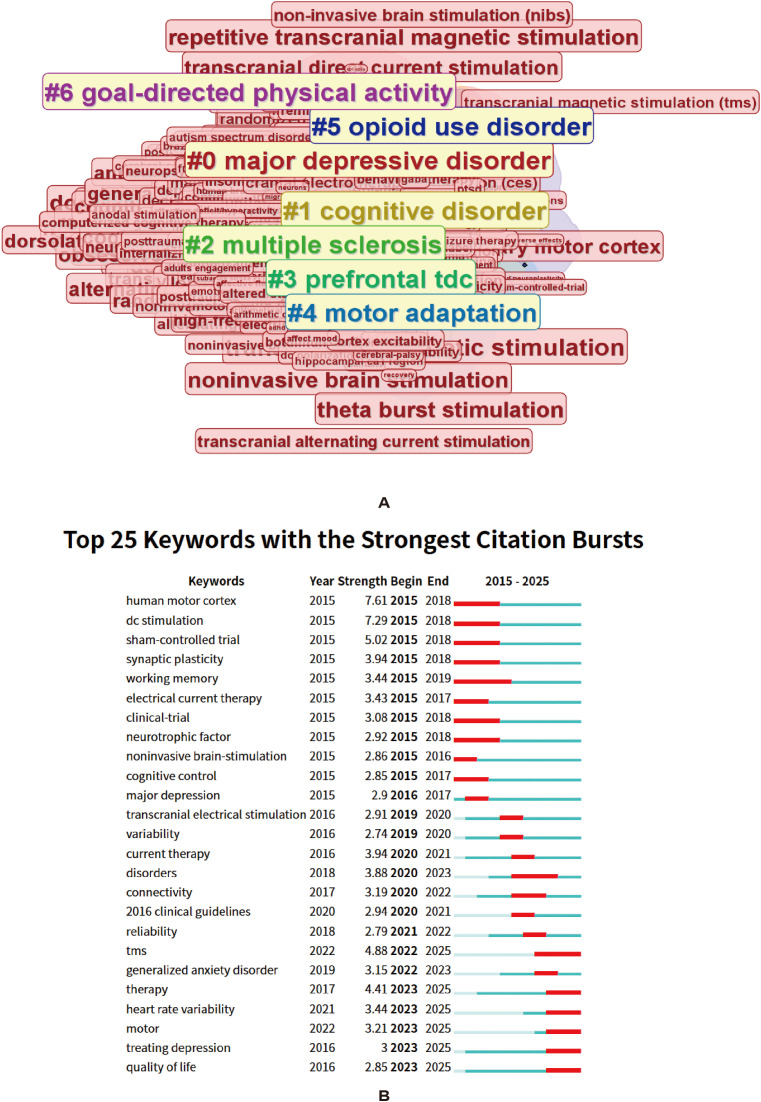
Recent thematic clusters and keyword citation bursts. **(A)** Shows the keyword cluster view for the recent period. **(B)** Shows the top 25 keywords with the strongest citation bursts during 2015–2025; the red segments indicate the periods of burst intensity. Colors indicate clusters generated by the algorithm. Interpretation relies on cluster labels, node proximity, and network structure rather than color differences alone.

**Figure 7:**
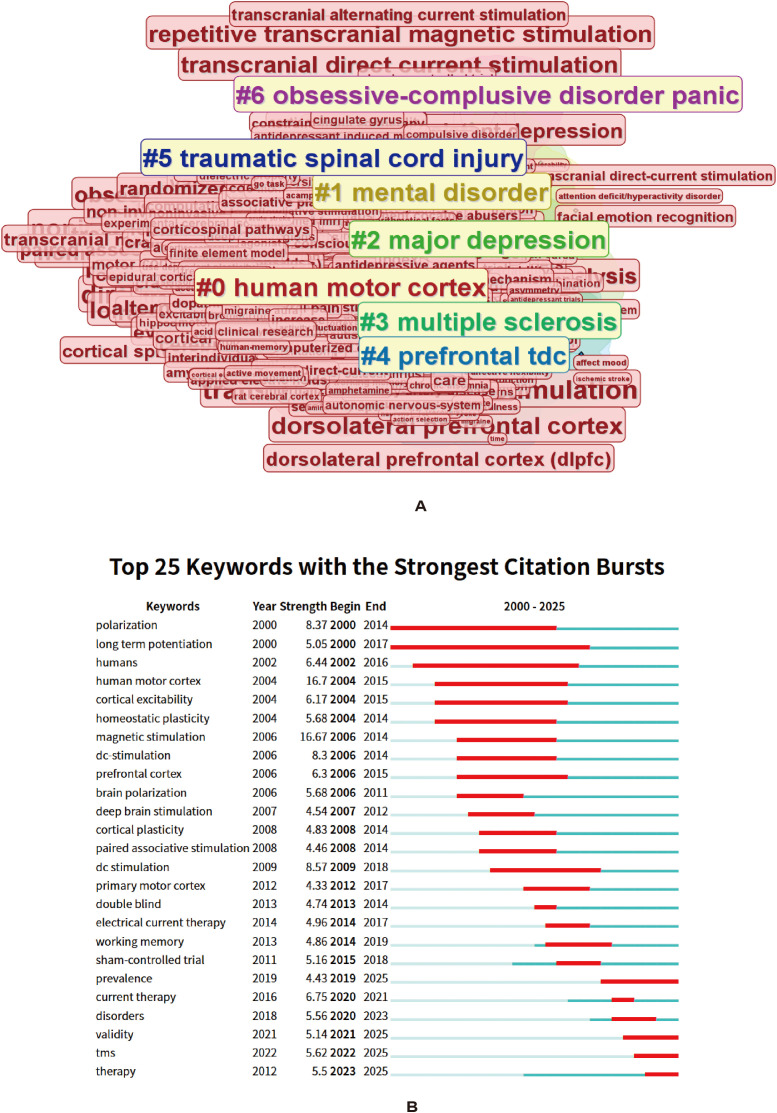
Full-span thematic clusters and keyword citation bursts. **(A)** Shows the keyword cluster view across the full study period. **(B)** Shows the top 25 keywords with the strongest citation bursts during 2000–2025; the red segments indicate the periods of burst intensity. Colors indicate clusters generated by the algorithm. Interpretation relies on cluster labels, node proximity, and network structure rather than color differences alone.

**Figures 6** and **7** present the keyword clustering results and burst-term analyses for the recent stage (2015–2025) and the overall stage (2000–2025), respectively.

1. Keyword clustering analysis: In the recent stage (**Figure 6A**, 2015–2025), keyword clustering revealed several relatively clear research themes. Cluster #0, “major depressive disorder”, was the largest, indicating that depression remained the core research topic in this field. Meanwhile, clusters such as #3 “prefrontal tDCS” and #4 “motor adaptation” suggest that research has focused on prefrontal-targeted stimulation and motor/functional regulation mechanisms. In addition, clusters #1 “cognitive disorder” and #2 “multiple sclerosis” indicate that the application of tDCS has gradually expanded to cognitive disorders and neurological diseases, whereas #5 “opioid use disorder” and #6 “goal-directed physical activity” reflect emerging extensions into addictive behaviors and behavioral regulation.

In the overall stage (**Figure 7A**, 2000–2025), the keyword clusters showed a broader and more foundational research structure. Core clusters included #0 “human motor cortex” and #4 “prefrontal tDCS”, reflecting that early studies mainly focused on neuromodulatory mechanisms involving the motor cortex and prefrontal cortex. In addition, #2 “major depression” and #1 “mental disorder” suggest that tDCS gradually became applied in psychiatric disorders, whereas #3 “multiple sclerosis” and #5 “traumatic spinal cord injury” reflect its extension into neurological disorders. By contrast, clusters such as #6 “obsessive-compulsive disorder panic” indicate a broader expansion of psychiatric research domains. Overall, from 2000–2025 to 2015–2025, research themes shifted from being primarily driven by neurophysiological mechanisms to being centered on clinical applications in depression, while also expanding toward multiple diseases and multidimensional functional regulation.

2. Burst keyword analysis.

Burst-term analysis (**Figures 6B**, **7B**) further revealed the temporal evolution of research hotspots. In the overall stage (2000–2025, **Figure 7B**), early burst terms were mainly concentrated in the domain of basic neural mechanisms, such as “polarization”, “long term potentiation”, “cortical excitability”, and “human motor cortex”, indicating that the field initially focused on neural plasticity and the physiological effects of electrical stimulation. Subsequently, research gradually shifted toward topics related to clinical study design, such as “double blind”, “sham-controlled trial”, and “working memory”, reflecting the introduction and development of evidence-based methodologies. In recent years, burst terms shifted toward “tms”, “therapy”, “validity”, and “disorders”, suggesting that research emphasis gradually turned to clinical application and efficacy evaluation.

In the recent stage (2015–2025, **Figure 6B**), burst terms displayed a more evident clinical and translational orientation. Early burst terms around 2015, such as “human motor cortex”, “dc stimulation”, and “synaptic plasticity”, were still mainly mechanism-related. More recently, especially after 2022, burst terms shifted toward “tms”, “therapy”, “quality of life”, “treating depression”, and “heart rate variability”, indicating that research focus has further expanded to clinical efficacy, quality of life, and physiological indicator assessment. At the same time, the emergence of terms such as “generalized anxiety disorder” also suggests that research targets have gradually expanded from depression alone to a broader spectrum of affective disorders.

Taken together, the clustering and burst-term analyses support a developmental pathway in tDCS research from exploration of neurophysiological mechanisms, to validation through clinical trials, and then toward expansion across multiple diseases and multidimensional outcomes. Current research is progressively moving toward precision intervention and multimodal integration.

### 3.8 Global and local citations

To further identify representative high-impact studies in the field of tDCS research on depression, this study separately analyzed the top 10 publications ranked by global citations (**Table 2**) and the top 10 ranked by local citations (**Table 3**). Among the globally cited studies (**Table 2**), the highest-ranked publication was Nitsche MA’s Excitability changes induced in the human motor cortex by weak, with 4,383 global citations, far exceeding all other publications, indicating its foundational influence in the broader field of neuromodulation and brain stimulation (25). It was followed by Lefaucheur JP’s 2017 evidence-based guidelines on the therapeutic application of tDCS (1,320 citations) (26) and Brunoni AR’s review on challenges and future directions in clinical tDCS research (1,082 citations) (27). In addition, Antal A’s guideline paper on the safety and ethical considerations of low-intensity transcranial electrical stimulation (967 citations) (28) and Poreisz C’s study on the safety of tDCS (851 citations) also ranked highly (29). Overall, the globally highly cited literature was mainly concentrated in three areas: fundamental neurophysiological mechanism research, clinical application and efficacy studies, and safety and standardization guidelines. This suggests that the most influential studies in the field have focused not only on therapeutic efficacy but also on stimulation mechanisms, safety boundaries, and standards for clinical implementation.

**Table 2:** Top 10 globally cited articles.

No.	First author	Article title	Journal	Year	Global citations
1	NITSCHE MA	Excitability changes induced in the human motor cortex by weak transcranial direct current stimulation	J PHYSIOL-LONDON	2000	4383
2	LEFAUCHEUR JP	Evidence-based guidelines on the therapeutic use of transcranial direct current stimulation (tDCS)	CLIN NEUROPHYSIOL	2017	1320
3	BRUNONI AR	Clinical research with transcranial direct current stimulation (tDCS): challenges and future directions	BRAIN STIMUL	2012	1082
4	ANTAL A	Low intensity transcranial electric stimulation: Safety, ethical, legal regulatory and application guidelines	CLIN NEUROPHYSIOL	2017	967
5	POREISZ C	Safety aspects of transcranial direct current stimulation concerning healthy subjects and patients	RES BULL	2007	851
6	AL-HARBI KS	Treatment-resistant depression: therapeutic trends, challenges, and future directions	PREFER ADHER	2012	607
7	SIEBNER HR	Preconditioning of low-frequency repetitive transcranial magnetic stimulation with transcranial direct current stimulation: evidence for homeostatic plasticity in the human motor cortex	J NEUROSCI	2004	586
8	ONG WY	Role of the Prefrontal Cortex in Pain Processing	MOL NEUROBIOL	2019	577
9	CHEERAN B	A common polymorphism in the brain-derived neurotrophic factor gene (BDNF) modulates human cortical plasticity and the response to rTMS	J PHYSIOL-LONDON	2008	576
10	FREGNI F	A sham-controlled, phase II trial of transcranial direct current stimulation for the treatment of central pain in traumatic spinal cord injury	PAIN	2006	555

Articles are ranked by global citation counts.

**Table 3:** Top 10 locally cited references.

No.	First author	Article title	Journal	Year	Local citations
1	NITSCHE MA	Excitability changes induced in the human motor cortex by weak transcranial direct current stimulation	J PHYSIOL-LONDON	2000	448
2	BRUNONI AR	The sertraline vs. electrical current therapy for treating depression clinical study: results from a factorial, randomized, controlled trial	JAMA PSYCHIAT	2013	263
3	LOO CK	Transcranial direct current stimulation for depression: 3-week, randomised, sham-controlled trial	J PSYCHIAT	2012	212
4	LEFAUCHEUR JP	Evidence-based guidelines on the therapeutic use of transcranial direct current stimulation (tDCS)	CLIN NEUROPHYSIOL	2017	206
5	BOGGIO PS	A randomized, double-blind clinical trial on the efficacy of cortical direct current stimulation for the treatment of major depression	J NEUROPSYCHOPH	2008	205
6	BRUNONI AR	Clinical research with transcranial direct current stimulation (tDCS): challenges and future directions	BRAIN STIMUL	2012	194
7	BRUNONI AR	Trial of Electrical Direct-Current Therapy versus Escitalopram for Depression	J MED	2017	166
8	BRUNONI AR	Transcranial direct current stimulation for acute major depressive episodes: meta-analysis of individual patient data	J PSYCHIAT	2016	155
9	LOO CK	A double-blind, sham-controlled trial of transcranial direct current stimulation for the treatment of depression	J NEUROPSYCHOPH	2010	135
10	KALU UG	Transcranial direct current stimulation in the treatment of major depression: a meta-analysis	PSYCHOL MED	2012	119

Local citations refer to citations received within the retrieved dataset.

Among the locally cited studies (**Table 3**), the highest-ranked publication was again Nitsche MA’s classic study, with 448 local citations, indicating that this publication was highly influential not only in the broader academic network but also within the tDCS-and-depression literature system included in this study (25). It was followed by Brunoni AR’s 2013 randomized controlled trial comparing sertraline versus electrical current therapy (263 local citations) (30) and Loo CK’s 2012 three-week randomized sham-controlled trial of tDCS for depression (212 local citations) (31). In addition, Boggio PS’s randomized double-blind trial of cortical direct current stimulation for major depression (205 citations) (32), Brunoni AR’s review on challenges and future directions in clinical tDCS research (194 citations) (27), and Kalu UG’s meta-analysis (119 citations) also ranked among the top 10 (33). Compared with the globally cited literature, the locally highly cited studies were more concentrated in clinical trials, randomized controlled studies, and meta-analyses on depression, indicating that within the field of tDCS research on depression, the most influential evidence mainly derives from pivotal clinical trials and evidence syntheses.

Taken together, **Tables 2** and **3** show that the top 10 globally cited and top 10 locally cited publications overlapped to some extent, while also reflecting different levels of influence. The former mainly reflects the overall academic impact of publications in the broader field of brain stimulation research, whereas the latter better reveals their knowledge contribution within the specific field of tDCS for depression. Overall, the knowledge base of this field has been established primarily on the work of representative scholars such as Nitsche, Brunoni, Loo, Boggio, and Lefaucheur, encompassing both foundational neurophysiological mechanism studies and key trials and guideline papers that have advanced clinical translation.

## 4 Discussion

This study conducted a systematic bibliometric analysis based on data from multiple databases to examine research on tDCS in the field of depression from 2000 to 2025. Overall, the field has shown sustained growth, with a marked acceleration in recent years. After 2006, publication output entered a phase of steady expansion, which further accelerated after 2020 and reached a peak in 2024. Although there was a slight decline in 2025, the overall publication volume remained at a relatively high level. Keyword co-occurrence analysis showed that current high-frequency themes are mainly concentrated in areas such as the prefrontal cortex/dlPFC, major depression, treatment outcome, anxiety, and cognition. These findings suggest that the research focus has shifted from the simple assessment of symptom improvement to more integrative investigations involving brain-targeted stimulation, functional outcomes, and underlying neural mechanisms. This transition is consistent with the developmental trajectory reflected in the highly cited literature. Early studies, exemplified by the work of Nitsche and colleagues, primarily focused on cortical excitability and basic neurophysiological mechanisms, whereas subsequent research gradually evolved into clinical studies and evidence-based guideline frameworks represented by Brunoni, Lefaucheur, and others. Together, this progression reflects the continuing deepening of the field from basic research toward clinical translation. In addition, analyses of country- and institution-level collaboration networks indicate that countries such as the United States, Germany, and Brazil occupy central positions in both publication output and collaborative networks. Most high-output countries also exhibit a high proportion of international collaboration, suggesting that the field has developed a relatively mature global collaborative framework.

### 4.1 Global landscape and research evolution: from local modulation to network integration

The sustained expansion of tDCS research in depression reflects both deepening conceptual understanding of neuromodulatory mechanisms and a notable shift in methodological orientation. Early work largely centered on feasibility, safety, and short-term clinical effects. In a seminal synthesis, Brunoni and colleagues highlighted the necessity of rigorous randomized, double-blind, sham-controlled designs for establishing clinical evidence and emphasized that tDCS effects were primarily mediated through modulation of local cortical excitability and plasticity (27). As evidence accrued, highly influential studies catalyzed a broader, systems-level interpretive framework. On the one hand, the evidence-based guideline by Lefaucheur et al. delineated key parameters, clinical boundaries, and safety specifications, establishing a standardization scaffold for cross-study comparability and clinical translation (26). On the other hand, an emerging body of integrative evidence indicates that depression is more aptly conceptualized as a disorder of large-scale network dysregulation, with aberrant coupling among the default mode network (DMN), salience network (SN), and executive control network (ECN) being linked to symptom dimensions (e.g., affective reactivity, cognitive control, motivational/reward processing), thereby motivating a shift from “local stimulation effects” to “network-level modulation” (11).

In parallel, mechanistic work increasingly integrates “stimulation parameters–electric field distribution–network effects–clinical response” into unified explanatory and computational models, repositioning tDCS from a traditional “local excitability tool” toward a system-level intervention capable of shaping network organization. For example, high-definition stimulation work suggests that stimulation can alter large-scale functional network organization (e.g., network segregation/integration profiles), indicating that effects are not confined to the immediate target region (10). Consistently, hybrid brain modeling studies propose that tDCS can influence whole-brain functional connectivity patterns and network efficiency, providing mechanistic support for a “parameter–network–phenotype” pathway (34). At the clinical evidence level, a parallel shift is evident: evidence syntheses increasingly emphasize the need to establish relative effectiveness across heterogeneous parameter configurations and to answer clinically actionable questions about “which protocols work best” rather than merely “whether tDCS works” (35). Meanwhile, response heterogeneity has become a field-wide constraint; multicenter randomized evidence indicates that identical protocols can yield divergent outcomes across patient groups, reinforcing the imperative to identify predictors grounded in baseline network states, comorbidity burden, and plasticity thresholds (13).

Accordingly, the field is increasingly converging on a system-level framework in which stimulation parameters, network coupling, and clinical phenotypes are jointly modeled to explain heterogeneity and enable precision optimization. This development trend is consistent with the bibliometric findings of the present study. On the one hand, annual publication output has shown sustained growth since 2006 and has accelerated markedly after 2020. On the other hand, relatively dense international collaboration networks have formed among high-output countries and institutions. In addition, keyword analysis highlights themes such as the prefrontal cortex/dlPFC, major depression, treatment outcome, anxiety, and cognition, suggesting that the research focus has gradually shifted from “whether it works” to “why it works, for whom it works, and under what conditions it works.” Finally, methodological expansion has been accompanied by increasing attention to implementation prerequisites. Expert guidelines emphasize the fundamental importance of safety training, ethical governance, and regulatory frameworks for ensuring reproducible and scalable neuromodulation research.

### 4.2 Thematic clusters and their scientific implications: toward multi-level integration

Keyword clustering indicates that tDCS–depression research has differentiated into several thematically coherent yet internally connected clusters. Rather than representing isolated lines of work, these clusters collectively delineate a translational arc linking network mechanisms, clinical-spectrum expansion, physiological calibration paradigms, and evidence upgrading. Overall, the knowledge structure of this field demonstrates a hierarchical pattern, encompassing both a vertical evolution from “mechanistic interpretation–clinical application–expansion to multiple disorders” and a horizontal integration across neuromodulation, psychiatry, and neuroscience, thereby providing a structured framework for understanding the migration of research hotspots. During the overall period (2000–2025), keyword clustering mainly included #0 “human motor cortex” #1 “mental disorder” #2 “major depression” #3 “multiple sclerosis” #4 “prefrontal tDCS” #5 “traumatic spinal cord injury” and #6 “obsessive-compulsive disorder panic”. This clustering pattern indicates that early research primarily focused on stimulation effects in the motor cortex and prefrontal cortex, underlying neurophysiological mechanisms, and their applications in neuropsychiatric disorders. In particular, “human motor cortex” and “prefrontal tDCS” suggest that the field has long emphasized fundamental stimulation effects and the modulation of key target regions. Classic studies have demonstrated that weak direct current applied through the scalp can noninvasively modulate motor cortical excitability, with anodal stimulation generally enhancing cortical excitability and cathodal stimulation generally reducing it; these effects may vary depending on stimulation intensity and duration. The underlying mechanisms may involve changes in membrane polarization and sustained neuroplastic processes following stimulation. These findings suggest that weak transcranial electrical stimulation is a noninvasive, reversible, and locally targeted approach to brain modulation, and they also provided an important physiological basis for its subsequent application in psychiatric disorders such as depression.

Meanwhile, subsequent reviews have further noted that as tDCS has progressed from basic research and early clinical trials toward more advanced stages of clinical translation, research priorities have expanded beyond its physiological effects to include stimulation parameter optimization, computational modeling, staged clinical trial design, and ethical and regulatory considerations (27). This indicates that the development of tDCS research has not followed a simple linear transition from “mechanistic research” to “clinical application” but has instead been accompanied by parallel advancements in methodology and implementation frameworks during the translational process. From this perspective, clusters such as “major depression” and “mental disorder” suggest that tDCS has gradually extended from fundamental neuromodulatory mechanism studies to psychiatric treatment and has increasingly entered the core domain of depression research. Meanwhile, clusters such as “multiple sclerosis” and “traumatic spinal cord injury” indicate that the exploration of tDCS in neurological disorders has not only broadened its clinical indications but also provided methodological and physiological references for its translational application in depression (31, 33). Overall, the clustering results in the overall period reflect a knowledge structure with a strong orientation toward fundamental mechanisms, while also demonstrating its gradual expansion toward psychiatric disorders and cross-disease applications.

In the recent period (2015–2025), the clustering structure further evolved into #0 “major depressive disorder” #1 “cognitive disorder” #2 “multiple sclerosis” #3 “prefrontal tDCS” #4 “motor adaptation” #5 “opioid use disorder” and #6 “goal-directed physical activity”. Compared with the overall period, recent clusters focus more directly on major depressive disorder itself and place greater emphasis on cognitive function, prefrontal-targeted stimulation, motor/functional adaptation, and behavioral regulation. This suggests that the research focus has shifted from early mechanistic exploration and cross-disease applications toward depression-centered clinical applications, while increasingly addressing emerging domains such as cognitive dysfunction, addictive behaviors, and multidimensional functional outcomes. This trend is consistent with directions highlighted in subsequent evidence-based guidelines and clinical summaries.

A relevant European expert consensus, based on systematic evaluation of repeated sham-controlled tDCS studies, indicated that tDCS research has progressed from early feasibility validation toward stages of indication stratification, efficacy grading, and parameter standardization. Specifically, anodal tDCS over the left DLPFC was considered to have a level B recommendation (probably effective) for non–drug-resistant major depressive episodes, whereas anodal stimulation over the right DLPFC showed a level B recommendation in the context of addiction/craving. In addition, applications for symptoms related to multiple sclerosis and spinal cord injury were also incorporated into the evidence evaluation framework (26). These findings indicate that themes such as “major depressive disorder”, “prefrontal tDCS”, “multiple sclerosis”, and “opioid use disorder” did not emerge in isolation, but rather reflect the ongoing refinement of tDCS research around specific indications, key stimulation targets, and clinically applicable protocols. At the same time, these guidelines also emphasize that current evidence remains insufficient for any indication to achieve the highest level of definitive efficacy recommendation, suggesting that, despite rapid progress toward clinical translation, issues such as treatment stability, parameter optimization, and patient stratification remain to be addressed.

Furthermore, given that tDCS devices are relatively easy to operate and cost-effective, they hold potential for home-based applications. Accordingly, research attention has increasingly expanded to implementation-related issues, including professional training, patient education, ethical governance, and regulatory frameworks. This indicates that, in the recent stage, research hotspots are reflected not only in the expansion of clinical indications but also in a shift from asking “whether it works” to addressing “how to implement it in a standardized manner”, “how to optimize its application”, and “how to ensure safe dissemination”.

Burst keyword analysis further supports the above evolutionary trajectory. In the overall period, early burst keywords were mainly concentrated in fundamental neurophysiological mechanism–related terms such as “polarization”, “long term potentiation”, “cortical excitability”, and “human motor cortex” (25). Overall, the knowledge structure of this field demonstrates a hierarchical pattern, encompassing both a vertical evolution from “mechanistic interpretation–clinical application–expansion to multiple disorders” and a horizontal integration across neuromodulation, psychiatry, and neuroscience, thereby providing a structured framework for understanding the migration of research hotspots. During the overall period (2000–2025), keyword clustering mainly included #0 “human motor cortex”, #1 “mental disorder”, #2 “major depression”, #3 “multiple sclerosis”, #4 “prefrontal tDCS”, #5 “traumatic spinal cord injury”, and #6 “obsessive-compulsive disorder panic”. This clustering pattern indicates that early research primarily focused on stimulation effects in the motor cortex and prefrontal cortex, underlying neurophysiological mechanisms, and their applications in neuropsychiatric disorders. In particular, “human motor cortex” and “prefrontal tDCS” suggest that the field has long emphasized fundamental stimulation effects and the modulation of key target regions. Classic studies have demonstrated that weak direct current applied through the scalp can noninvasively modulate motor cortical excitability, with anodal stimulation generally enhancing cortical excitability and cathodal stimulation generally reducing it; these effects may vary depending on stimulation intensity and duration. The underlying mechanisms may involve changes in membrane polarization and sustained neuroplastic processes following stimulation. These findings suggest that weak transcranial electrical stimulation is a noninvasive, reversible, and locally targeted approach to brain modulation, and they also provided an important physiological basis for its subsequent application in psychiatric disorders such as depression (25).

Meanwhile, subsequent reviews have further noted that as tDCS has progressed from basic research and early clinical trials toward more advanced stages of clinical translation, research priorities have expanded beyond its physiological effects to include stimulation parameter optimization, computational modeling, staged clinical trial design, and ethical and regulatory considerations (27). This indicates that the development of tDCS research has not followed a simple linear transition from “mechanistic research” to “clinical application”, but has instead been accompanied by parallel advancements in methodology and implementation frameworks during the translational process. From this perspective, clusters such as “major depression” and “mental disorder” suggest that tDCS has gradually extended from fundamental neuromodulatory mechanism studies to psychiatric treatment and has increasingly entered the core domain of depression research. Meanwhile, clusters such as “multiple sclerosis” and “traumatic spinal cord injury” indicate that the exploration of tDCS in neurological disorders has not only broadened its clinical indications but also provided methodological and physiological references for its translational application in depression (31, 33). Overall, the clustering results in the overall period reflect a knowledge structure with a strong orientation toward fundamental mechanisms, while also demonstrating its gradual expansion toward psychiatric disorders and cross-disease applications.

In the recent period (2015–2025), the clustering structure further evolved into #0 “major depressive disorder”, #1 “cognitive disorder”, #2 “multiple sclerosis”, #3 “prefrontal tDCS”, #4 “motor adaptation”, #5 “opioid use disorder”, and #6 “goal-directed physical activity”. Compared with the overall period, recent clusters focus more directly on major depressive disorder itself and place greater emphasis on cognitive function, prefrontal-targeted stimulation, motor/functional adaptation, and behavioral regulation. This suggests that the research focus has shifted from early mechanistic exploration and cross-disease applications toward depression-centered clinical applications, while increasingly addressing emerging domains such as cognitive dysfunction, addictive behaviors, and multidimensional functional outcomes. This trend is consistent with directions highlighted in subsequent evidence-based guidelines and clinical summaries.

A relevant European expert consensus, based on systematic evaluation of repeated sham-controlled tDCS studies, indicated that tDCS research has progressed from early feasibility validation toward stages of indication stratification, efficacy grading, and parameter standardization. Specifically, anodal tDCS over the left DLPFC was considered to have a level B recommendation (probably effective) for non–drug-resistant major depressive episodes, whereas anodal stimulation over the right DLPFC showed a level B recommendation in the context of addiction/craving. In addition, applications for symptoms related to multiple sclerosis and spinal cord injury were also incorporated into the evidence evaluation framework (26). These findings indicate that themes such as “major depressive disorder”, “prefrontal tDCS”, “multiple sclerosis”, and “opioid use disorder” did not emerge in isolation, but rather reflect the ongoing refinement of tDCS research around specific indications, key stimulation targets, and clinically applicable protocols. At the same time, these guidelines also emphasize that current evidence remains insufficient for any indication to achieve the highest level of definitive efficacy recommendation, suggesting that despite rapid progress toward clinical translation, issues such as treatment stability, parameter optimization, and patient stratification remain to be addressed.

Furthermore, given that tDCS devices are relatively easy to operate and cost-effective, they hold potential for home-based applications. Accordingly, research attention has increasingly expanded to implementation-related issues, including professional training, patient education, ethical governance, and regulatory frameworks. This indicates that, in the recent stage, research hotspots are reflected not only in the expansion of clinical indications but also in a shift from asking “whether it works” to addressing “how to implement it in a standardized manner”, “how to optimize its application”, and “how to ensure safe dissemination”.

Burst keyword analysis further supports the above evolutionary pathway. In the overall period, early burst terms were mainly concentrated in fundamental neurophysiological mechanism-related terms such as “polarization”, “long term potentiation”, “cortical excitability”, and “human motor cortex”, indicating that the field initially focused primarily on the regulatory effects of tDCS on cortical excitability and neuroplasticity. Subsequently, research hotspots gradually shifted toward terms related to clinical study design and functional outcomes, such as “double blind”, “sham-controlled trial”, and “working memory” which is consistent with the increasing number of randomized, double-blind, sham-controlled trials in depression. Related studies have shown that tDCS in depression treatment has begun to validate its efficacy through more rigorous trial designs, while also paying greater attention to broader clinical outcomes such as cognitive function (31, 36). More recent individual patient data meta-analyses have further shown that although tDCS produces a small but statistically significant improvement in depressive symptoms, the overall effect size remains limited and there is still some heterogeneity among studies, suggesting that the focus of the field has gradually shifted from early mechanistic validation to efficacy evaluation, methodological optimization, and the identification of factors influencing treatment response. In recent years, terms such as “tms”, “therapy”, “validity”, and “disorders” have emerged as representative burst terms, indicating that research has increasingly focused on clinical application and efficacy evaluation (37). In the recent stage, burst terms more clearly reflect clinical and translational characteristics. In addition to mechanistic terms such as “human motor cortex”, “dc stimulation”, and “synaptic plasticity”, the recent appearance of terms such as “quality of life”, “treating depression”, “heart rate variability”, and “generalized anxiety disorder” suggests that both the research population and the observed outcome measures are expanding (25). Overall, these clusters and burst terms jointly indicate that tDCS-related research has continuously evolved along a trajectory from “exploration of neurophysiological mechanisms” to “validation through clinical trials” and then to “expansion across multiple disorders and multidimensional functional outcomes,” and is gradually moving toward a new stage characterized by precision intervention, multimodal integration, and real-world application. It should be noted that the appearance of non-depression-related themes such as “multiple sclerosis” and “opioid use disorder” in both the recent and overall clustering stages more likely reflects the methodological and mechanistic commonalities of non-invasive brain stimulation research across different neuropsychiatric disorders, rather than a shift in the thematic focus of the present study.

It is also worth noting that although the present study focused on tDCS, a considerable number of terms related to TMS still appeared in the keyword co-occurrence and burst analyses. This more likely reflects the substantial overlap among non-invasive brain stimulation techniques in terms of stimulation targets, study design, and mechanistic exploration, rather than indicating a shift in research focus from tDCS to TMS.

### 4.3 Clinical and translational implications: from evidence standards to stratified decision-making and implementability

The knowledge evolution captured here—from early efficacy validation to network-based mechanistic inquiry and evidence integration—highlights both persistent bottlenecks and plausible pathways forward. Based on current bibliometric characteristics, future progress will likely depend on the following priorities.

First, response heterogeneity remains the dominant obstacle. The sustained rise of “functional connectivity” suggests that undifferentiated, “one-size-fits-all” trial designs may increasingly underperform by obscuring true effects within biologically heterogeneous samples (38). Multicenter randomized evidence demonstrates substantial variability in response even under tightly controlled conditions (13). Accordingly, future high-quality studies should shift toward stratified designs, incorporating baseline network signatures (e.g., prefrontal–limbic coupling strength) and plasticity-related phenotypes into predictive models to identify likely responders. Such data-driven stratification directly addresses “for whom does it work,” and mitigates false-negative conclusions driven by heterogeneity (39).

Second, evidence quality and generalizability remain constrained by protocol reporting variability. Although the “human motor cortex” cluster underscores attention to physiological benchmarking, heterogeneity in reporting clinically salient parameters (e.g., montage, current density, and sham-blinding procedures) continues to compromise reproducibility (27, 40). While guidelines provide foundational specifications, consistent implementation across multicenter studies remains an open challenge (26). Building a standardized, multicenter evidence ecosystem is therefore urgent, including harmonized core outcome sets and data-sharing protocols, enabling large-scale synthesis and reducing single-center noise.

Third, the rise of “network meta-analysis” reflects a clear shift in clinical demand: from evaluating tDCS in isolation to establishing its relative positioning within the broader antidepressant intervention landscape (41). Contemporary evidence syntheses emphasize the need to resolve comparative effectiveness across complex parameter regimes (35). Progress will require more high-quality head-to-head comparative trials to clarify the niche of tDCS relative to rTMS, TBS, and pharmacotherapy—e.g., whether it is best positioned as a stand-alone first-line option for mild-to-moderate depression or as an augmentation strategy in treatment-resistant cases (30, 42).

Finally, rapid growth in citations related to home-based tDCS highlights distinct translational potential associated with portability and decentralization (18). Recent randomized trials indicate that, under stringent remote-monitoring protocols, home-delivered treatment may show encouraging profiles in maintenance efficacy and adherence (4, 18). This trend suggests that the research agenda should increasingly incorporate implementation science—addressing remote supervision, long-term maintenance strategies, and cost-effectiveness—so that tDCS accessibility advantages can translate into real-world public health benefit.

### 4.4 Limitations and future directions

Several caveats warrant a cautious interpretation of our findings. Although multi-database integration improves coverage, inherent differences across databases in indexing criteria, metadata structures, and field definitions may introduce residual noise into co-occurrence networks and clustering stability. In addition, citation-based indicators are subject to time-lag bias, potentially underrepresenting genuinely influential yet recently emerging concepts or technologies that have not had sufficient time to accrue citations. The restriction to English-language publications may introduce language bias. Although this strategy is common in bibliometric studies and helps ensure consistency in retrieval and analysis, it may lead to the omission of important studies published in other languages. Therefore, the findings of this study should be interpreted with caution. Most importantly, bibliometrics provides a macroscopic map of knowledge topology; while it can robustly characterize hotspot migration and intellectual structure, it cannot substitute for micro-level causal validation of mechanisms or direct estimation of clinical effect magnitude.

Future progress will benefit from combining quantitative science mapping with targeted qualitative synthesis. Specifically, three directions appear particularly salient: (i) causal validation—moving beyond correlational network descriptions to test the causal specificity and reproducibility of network mechanisms in tDCS; (ii) ecosystem building—establishing multicenter, standardized, shared-data infrastructures that integrate imaging, physiology, behavior, and clinical outcomes to support stratification and prediction; and (iii) closed-loop evidence—conducting more rigorous head-to-head comparisons across key parameter and course configurations to generate an actionable evidence loop capable of guiding clinical decisions. Overall, the thematic reconfiguration and evidence upgrading observed here suggest that tDCS–depression research is moving beyond a phase of simple accumulation toward deeper mechanistic refinement and higher-order evidence integration.

## 5 Conclusion

Using data from the, Scopus, and PubMed, this study applied bibliometrix, VOSviewer, and CiteSpace to conduct a bibliometric and science-mapping analysis of research on tDCS and depression, thereby characterizing the field’s global landscape and temporal evolution. The results showed that, from 2000 to 2025, research output in this field increased steadily, with a marked acceleration in recent years, suggesting that tDCS has gradually evolved from an exploratory topic into an important theme in neuromodulation research. Meanwhile, the research focus has shifted from early validation of efficacy and safety toward a multilayered framework centered on prefrontal-targeted stimulation, clinical efficacy evaluation, mechanistic interpretation, and functional outcomes, reflecting a broader transition from “local stimulation effects” to “system-level integration and precision intervention.” Analyses of keyword frequency, co-occurrence networks, clustering, and burst terms indicate that the field currently focuses on several main directions, including studies of key stimulation targets such as the prefrontal cortex and dlPFC, evaluation of clinical efficacy and treatment outcomes in depression, investigation of neural mechanisms involving cognition, brain networks, and functional connectivity, and gradual expansion into multiple diseases and transdiagnostic contexts. The continued prominence of functional connectivity, cognitive outcomes, and prefrontal-targeted modulation suggests a shift from simple symptom evaluation toward linking brain network features, stimulation parameters, and clinical responses to improve patient stratification and treatment-response prediction. In addition, collaboration networks among countries, institutions, and authors show that the field has developed an international collaborative structure centered on highly productive countries such as the United States, Germany, and Brazil, as well as core institutions including the University of São Paulo, the University of Toronto, and the Harvard system. Highly cited publications further indicate that the knowledge base of the field is primarily grounded in fundamental neurophysiological mechanism studies, pivotal clinical trials, and evidence-based guidelines. Although the present study is constrained by differences in database coverage, language and time-window restrictions, and inevitable integration-related noise, the overall trajectory points toward mechanistic granularity and system-level synthesis. Future work should prioritize standardized, multicenter, multimodal designs with stratification by age/sex and clinical subtypes, and should integrate peripheral biomarkers and artificial intelligence–enabled analytics to facilitate translation of candidate markers into scalable clinical readouts and evidence-based decision support.

## Data Availability

The original contributions presented in the study are included in the article/**Supplementary Material**. Further inquiries can be directed to the corresponding author.
